# Lumbar disc herniation is an independent predictor of plaque burden in the patients with unstable angina

**DOI:** 10.3389/fcvm.2024.1324456

**Published:** 2024-02-09

**Authors:** Yongchao Wang, Junhua Ge, Hao Xu, Jian Li

**Affiliations:** Department of Cardiology, The Affiliated Hospital of Qingdao University, Qingdao, Shandong, China

**Keywords:** unstable angina, Gensini score, plaque burden, lumbar disc herniation, evolocumab treatment

## Abstract

**Objective:**

Assessing the impact of lumbar disc herniation (LDH) on the plaque burden of coronary atherosclerosis is our objective.

**Methods:**

In this study, a total of 212 patients (age 46–80 years) with unstable angina (UA) who underwent coronary angiography (CAG) in our hospital from January 2018 to July 2022 due to UA were included. Patients were divided into LDH group (*n* = 106) and no LDH group (*n* = 106). Gensini scores were calculated to assess the plaque burden of coronary. Logistic analysis was used to examine potential risk variables linked to the Gensini score. The association between lumbar disc lesions grading and coronary plaque burden was analysed by Spearman's correlation test. LDH patients with higher plaque burden (*n* = 56) were further divided into evolocumab treatment group (*n* = 28) and conventional treatment group (*n* = 28). Cox regression analysis were performed.

**Results:**

Patients with LDH had higher Gensini scores (*P* < 0.01) and triglyceride (TG) levels (*P* = 0.04), but HDL-C (*P* = 0.01) levels were lower. LDH was found to be an independent risk factor for higher Gensini scores (OR = 2.38, *P* < 0.01) by logistic analysis. The Spearman's correlation test suggested that the degree of lumbar disc lesions was related to the Gensini score and the level of blood lipid. Cox regression analysis showed that evolocumab treatment could significantly reduce the composite MACE events (cardiac death, nonfatal myocardial infarction, nonfatal stroke, and readmission due to angina) (HR = 0.26, *P* = 0.04) in higher coronary plaque burden patients.

**Conclusion:**

LDH is an independent risk factor for the higher coronary plaque burden. Evolocumab treatment significantly reduced the occurrence of cardiovascular events in LDH patients with higher plaque burden. Additionally, our data indicate that LDH is associated with increased blood lipid, which may contribute to the development of plaque burden.

## 1 Introduction

The relationship between lumbar disc herniation (LDH) and atherosclerosis has attracted increasing attention in recent years (1). Previous studies have shown that cardiovascular risk factors, particularly smoking, are significantly and independently associated with symptomatic LDH (2). Moreover, Yang et al. (3) demonstrated that the prevalence of cardiovascular risks (evaluated by checking the presence of metabolic syndrome) in patients with degenerative lumbar spinal disease was relatively higher compared to that in the general population. Moreover, Zhu et al. (4) found that frequent back pain (*n* = 39, 10.3% vs. *n* = 42, 5.9%, HR = 1.78) and daily back pain (*n* = 34, 11.8% vs. *n* = 42, 5.9%, HR = 2.13) were related to higher risk of coronary heart event compared to infrequent back pain. According to the research of Beckworth et al. (5), lumbar disc degeneration is related to higher prevalence of aortic atherosclerosis (*n* = 11, 22.45% vs. *n* = 62, 87.32%, *P* < 0.01) and severe aortic atherosclerosis (*n* = 4, 8.16% vs. *n* = 58, 81.69%, *P* < 0.01).

Recently, many cardiovascular risk factors were found to be related to the coronary plaque burden. Yang et al. (6) indicated that high-risk plaques were more common in patients with metabolic syndrome. Systemic lupus erythematosus (7) and severe obstructive sleep apnea (8) were also found to be associated with significant coronary artery plaque burden. Unfortunately, there are still no studies about the relationship between LDH and the plaque burden of coronary artery. To address this issue, the main goal of the present study was to evaluate the relationship between LDH and plaque burden in unstable angina (UA) patients.

## 2 Methods

### 2.1 Definitions

UA was defined based on 2021 AHA/ACC/ASE/CHEST/SAEM/SCCT/SCMR Guideline for the Evaluation and Diagnosis of Chest Pain (9). LDH was defined as the displacement of the nucleus pulposus of the intervertebral disc through the annulus fibrosus, thereby causing pressure on the neural elements (10). The diagnosis methods including symptoms (back pain and associated leg symptoms), physical examination (the straight leg raise test, the contralateral straight leg raise test, the femoral nerve stretch test, and the bowstring sign) and MRI. The diagnosis of LDH was made by professional spinal surgeons. All patients included in the LDH group have two years or more history of lumbar disc herniation. Gensini score (11) is a widely used angiographic scoring system for quantifying the severity of cardiovascular atherosclerotic disease. We used Gensini score to evaluate coronary plaque burden in our study. The Pfirrmann grading system (12) and the grading system of migrated lumbar disc herniation (13) are both systems for grading the degree of lumbar disc lesions of patients according to the patients' lumbar MRI image. We used the two grading systems to evaluate the degree of LDH in our study. Evolocumab treatment was defined as receiving 140 mg of evolocumab once every two weeks or 420 mg of evolocumab once a month for at least six months. Dyslipidaemia was defined based on 2019 ESC/EAS Guidelines for the management of dyslipidaemias (14). End-stage heart failure was defined as New York Heart Association class III-IV, despite optimal medical and device therapy, intolerance or withdrawal of evidence-based heart failure medications such as beta blockers, angiotensin converting enzyme inhibitors due to hypotension, diuretic-refractory volume overload, or need for intravenous inotrope. Severe hepatic dysfunction was defined as an increase in bilirubin that exceeded 10 times the normal value, prothrombin activity < 40%, or the presence of hepatic encephalopathy. Severe renal insufficiency is defined as estimated glomerular filtration rate less than 30 ml/min·1.73 m^2^.

### 2.2 Study population

**Figure 1:**
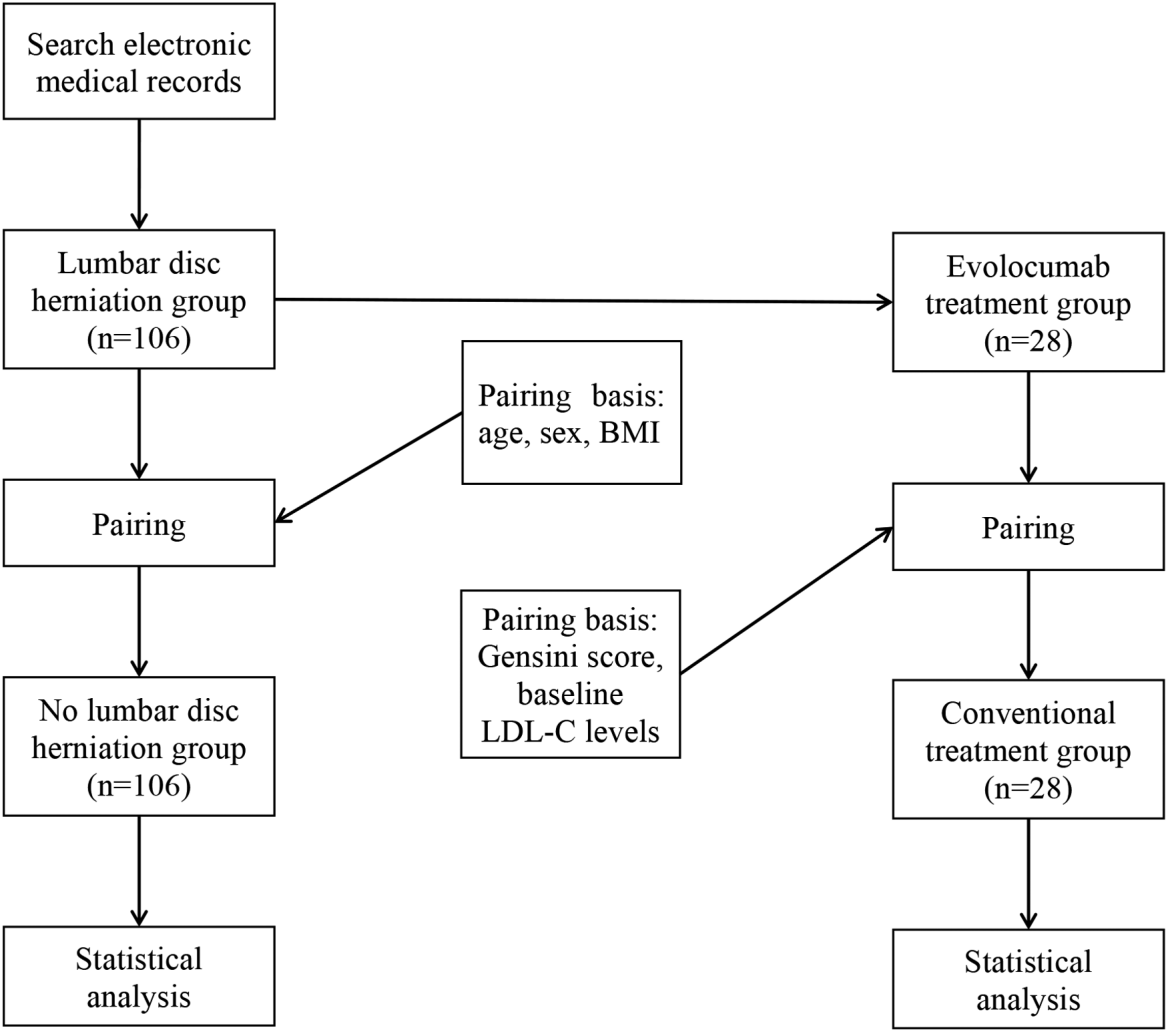
Study flowchart. BMI, weight and body mass index; LDL-C, low-density lipoprotein cholesterol.

The study was approved by the Affiliated Hospital of Qingdao University research ethics committee (No: QYFYWZLL28122), and all patients gave written informed consent to participate. Present study enrolled 212 UA patients (aged 46–80 years). First, we consecutively enrolled patients with UA and LDH, who were hospitalized in our hospital between January 2018 and July 2022 and underwent coronary angiography (CAG) to LDH group (*n* = 106). Then, no LDH group (*n* = 106) was matched for age, sex and BMI, consisted of UA patients, who were hospitalized in our hospital between January 2018 and July 2022 and underwent CAG. All patients received standard medical therapy. Patients in LDH group who received evolocumab treatment were included in the evolocumab treatment group (*n* = 28), and patients in LDH group who did not receive evolocumab treatment were matched into the conventional treatment group (*n* = 28) based on Gensini score and baseline low-density lipoprotein cholesterol level. All patients included in the evolocumab treatment group and conventional treatment group received basic statin treatment and their Gensini scores were higher than the first tertile of the LDH group.The rest of the treatment drugs were used in accordance with the current guidelines, and there were no potential differences in medical therapy between the two groups. Exclusion criteria were as follows: (1) age more than 80 years, (2) a history of revascularization, (3) end-stage heart failure, (4) a history of myocardial infarction, and (5) severe liver and kidney dysfunction. Figure 1, shows the study flowchart.

### 2.3 PCSK9 inhibitor treatment

LDL receptors play a role in the removal of circulating LDL-C from blood. When the PCSK9 protein binds to the LDL receptor, it starts the process of degrading the receptor, thus increasing LDL-C levels. The PCSK9 inhibitor inhibits PCSK9 binding to LDL receptors, increase recycling LDL receptors, and indirectly lower circulating LDL-C levels by increasing LDL-C uptake (15). For patients who have not achieved their lipid targets with statins in combination with ezetimibe, the latest ESC/EAS guidelines for the management of dyslipidaemia recommend advocating the use of PCSK9 inhibitor (14).

### 2.4 Study endpoint

The follow-up time for subgroup analysis was 1 year. End event was defined as major adverse cardiovascular events (MACEs) including cardiac death, nonfatal myocardial infarction, nonfatal stroke, and readmission due to angina.

### 2.5 Statistical processing

The characteristics of the patients are presented as the mean ± SD or interquartile range for continuous variables and were compared by paired Student's t test if the data were normally distributed; otherwise, the paired Wilcoxon signed rank test was used. Categorical variables are presented as percentages and were compared by Pearson's chi-square test. The association between lumbar disc lesions grading and coronary plaque burden was analysed by Spearman's correlation test. Age, sex and weight and body mass index (BMI) were control variables to expel their influence on the correlation coefficients. Clinical characteristics were all initially tested with univariate logistic analysis, and all variables with a *P* value < 0.1 were considered for inclusion in the multivariate model to investigate potential risk variables linked to higher Gensini score. Forest plots were drawn based on logistic analysis. Cox regression analysis and survival curve were used to evaluate the impact of the indicators on the outcomes of patients. All statistical tests were two-tailed, and *P* < 0.05 was considered to be statistically significant. All statistical analysis were carried out using the SPSS 26.0 software.

## 3 Results

### 3.1 Patients characteristics

A total of 212 UA patients were assessed. Baseline clinical characteristics of patients are summarized in Table 1. General characteristics, total cholesterol, LDL-C, glucose, hepatic aminotransferases levels and cardiac ultrasound indices were similar between two groups.

**Table 1:** Baseline data of lumbar disc herniation group and no lumbar disc herniation group.

	Lumbar disc herniation group	No lumbar disc herniation group	*P* value
	(*n* = 106)	(*n* = 106)	
Requiring revascularization	57 (53.77%)	32 (30.19%)	<0.01^c^
Gensini score	26.5 (10, 50.50)	14.0 (2, 37.25)	<0.01^c^
General characteristics			
Male (%)	57 (53.77%)	57 (53.77%)	-
Age (year)	66.43 ± 7.49	66.54 ± 7.34	-
BMI^b^ (kg/m^2^)	25.87 ± 3.23	25.11 ± 3.31	-
Hypertension (%)	74 (69.81%)	60 (56.60%)	0.06
Diabetes mellitus (%)	29 (27.36%)	23 (21.70%)	0.43
Drinking history (%)	22 (20.75%)	14 (13.21%)	0.20
Smoking history (%)	36 (33.96%)	23 (21.70%)	0.07
Laboratory test			
INR^b^	1.02 (0.95, 1.07)	1.04 (0.96, 1.11)	0.06
Fibrinogen (g/L)	3.05 (2.55, 3.42)	2.86 (2.49, 3.46)	0.44
D-dimer (ng/ml)	270 (180, 430)	330 (210, 410)	0.28
ALT^b^ (U/L)	18 (14, 27)	20 (15, 30)	0.41
AST^b^ (U/L)	18 (16, 25)	21 (18, 30)	0.05
Triglyceride (mmol/L)	1.33 (1.00, 1.94)	1.23 (0.83, 1.65)	0.04^c^
Total cholesterol (mmol/L)	4.57 ± 1.14	4.46 ± 1.18	0.96
HDL-C^b^ (mmol/L)	1.18 (1.05, 1.45)	1.31 (1.17, 1.54)	0.01^c^
LDL-C^b^ (mmol/L)	2.68 (1.99, 3.29)	2.56 (1.88, 3.09)	0.20
Lipoprotein A (mg/L)	187 (98, 339)	157 (60, 333)	0.63
eGFR^b^ (ml/min·1.73 m^2^)	69.33 (55.46, 85.00)	72.00 (55.5, 92.51)	0.51
Blood glucose (mmol/L)	5.55 (4.85, 6.67)	5.44 (4.99, 6.19)	0.56
Uric acid (umol/L)	348 (290, 407)	352 (299, 404)	0.90
Potassium ion (mmol/L)	4.07 (3.76, 4.38)	4.06 (3.90, 4.32)	0.87
Sodium ion (mmol/L)	142 (140, 143)	142 (141, 144)	0.60
Cardiac ultrasound indices			
LVIDd^b^ (cm)	4.7 (4.4, 4.9)	4.7 (4.4, 5.0)	0.33
LVIDs^b^ (cm)	3.1 (2.9, 3.2)	3 (2.8, 3.4)	0.72
LVEF^b^ (%)	62 (60, 64)	61 (59, 63)	0.07

^a^ Data are presented as the mean ± SD, media (Q_1_, Q_3_) or *n* (%).

^b^ BMI, weight and body mass index; INR, international normalized ratio; ALT, alanine aminotransferase; AST, aspartate aminotransferase; HDL-C, high-density lipoprotein cholesterol; LDL-C, low-density lipoprotein cholesterol; eGFR, estimated glomerular filtration rate; LVIDd, left ventricular internal diameter at end-diastole; LVIDs, left ventricular internal diameter at end-systole; LVEF, left ventricular ejection fraction.

^c^ *P* < 0.05, which means statistically significant.

As shown in Table 1, TG (median: 1.33 vs. 1.23 mmol/L, *p* = 0.04) levels were higher in the LDH group, while HDL-C (median: 1.18 vs. 1.31 mmol/L, *p* = 0.01) levels were lower. Our study suggested that the proportion of patients requiring coronary artery bypass graft surgery or percutaneous coronary intervention (57 vs. 32, *p* < 0.01) was higher in LDH group. The coronary plaque burden (median Gensini score: 26.5 vs. 14, *p* < 0.01) was also higher in LDH group.

**Figure 2:**
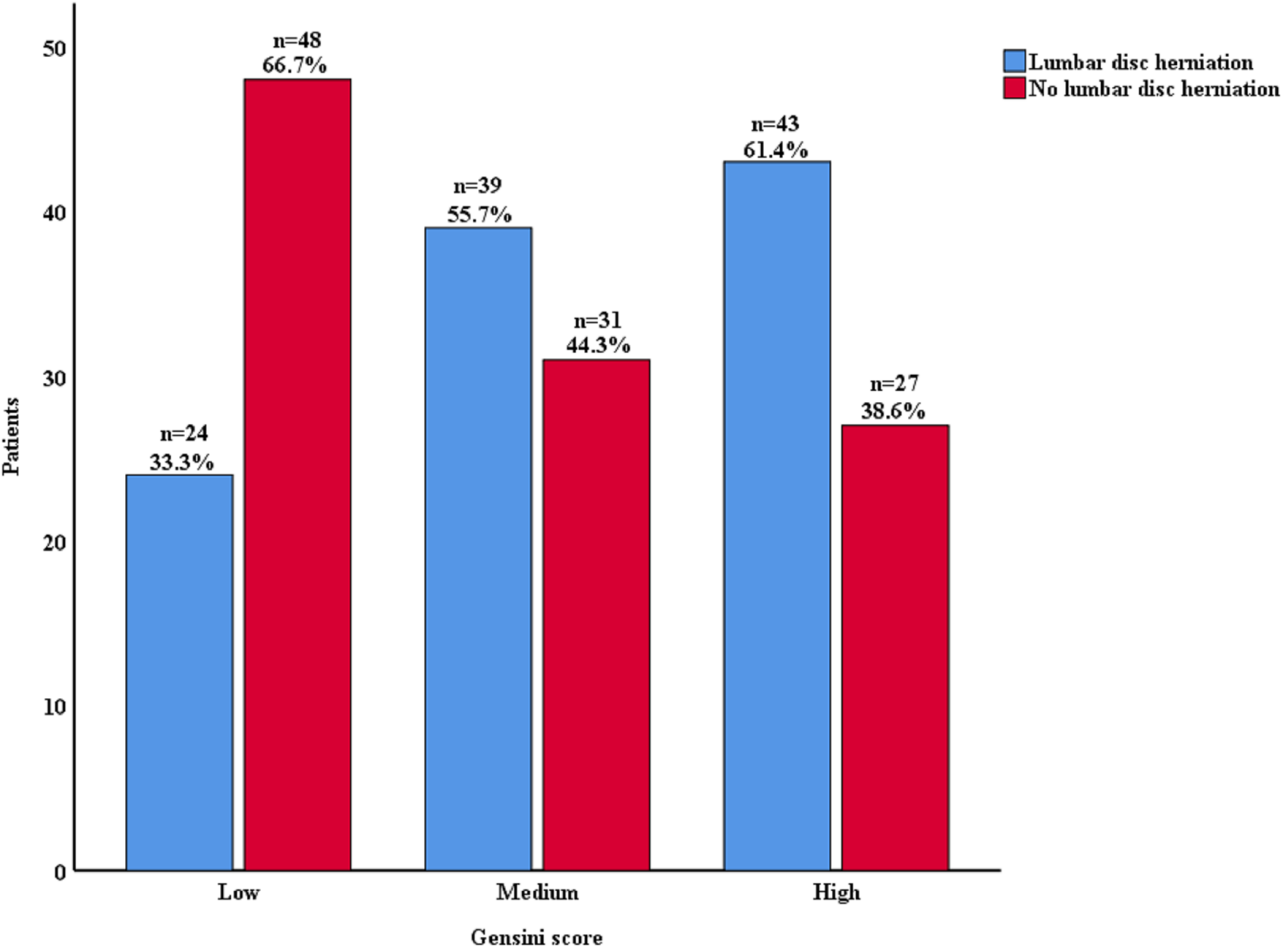
Lumbar disc herniation proportion according to Gensini score. Gensini score was divided by tertiles.

Figure 2 showed with the increase of Gensini score (divided by tertiles), the proportion of LDH also increased (33.3%, 55.7%, 61.4%).

### 3.2 Logistic analysis

**Figure 3:**
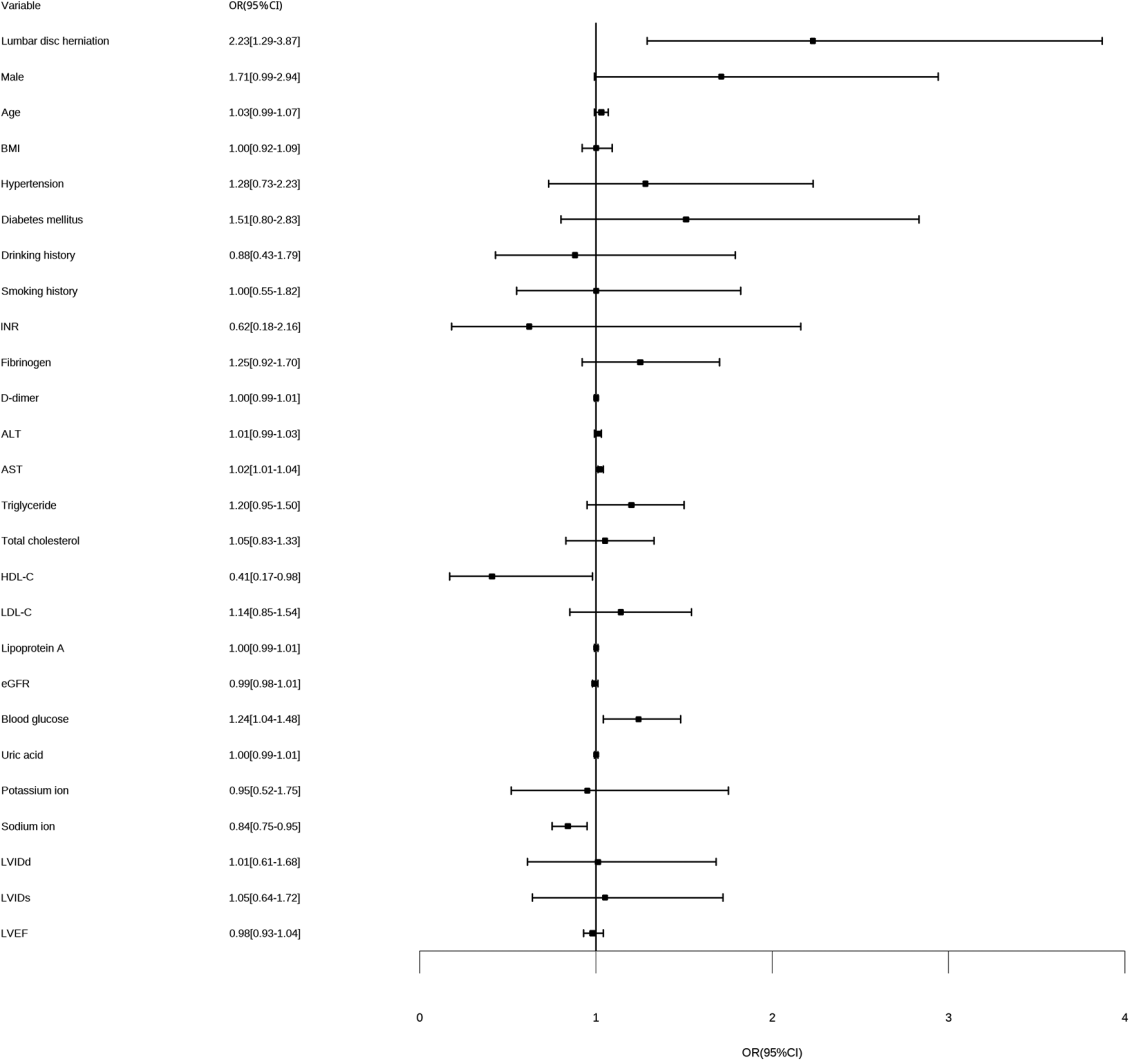
Forest plot for univariate logistic analysis. BMI, weight and body mass index; INR, international normalized ratio; ALT, alanine aminotransferase; AST, aspartate aminotransferase; HDL-C, high-density lipoprotein cholesterol; LDL-C, low-density lipoprotein cholesterol; eGFR, estimated glomerular filtration rate; LVIDd, left ventricular internal diameter at end-diastole; LVIDs, left ventricular internal diameter at end-systole; LVEF, left ventricular ejection fraction; OR, odds ratio for higher Gensini score.

**Figure 4:**
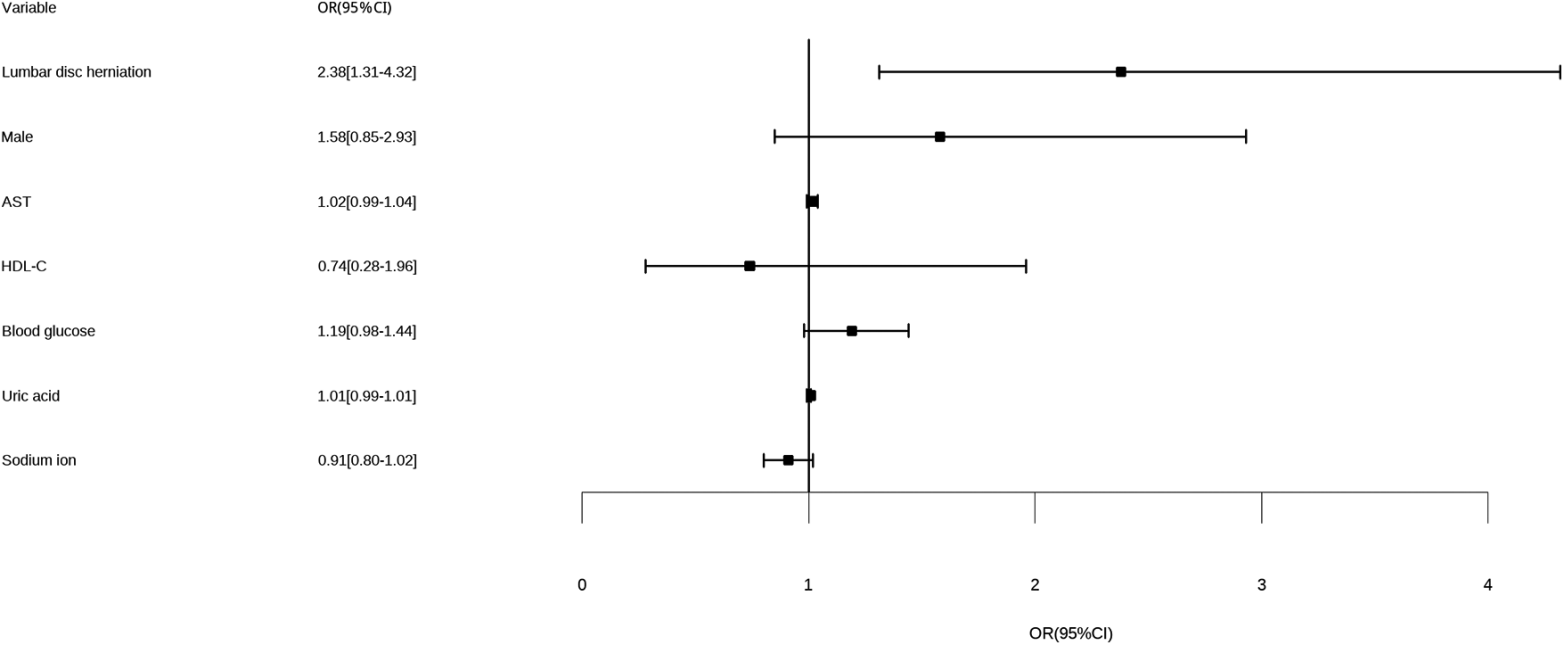
Forest plot for multivariate logistic analysis. AST, aspartate aminotransferase; HDL-C, high-density lipoprotein cholesterol; OR, odds ratio for higher Gensini score.

As shown in Table 2, Figures 3, 4, on multivariate model, LDH was found to be an independent risk factor for higher Gensini scores (OR = 2.38, 95% CI: 1.31–4.32, *p* < 0.01).

**Table 2:** Logistic analysis of risk factors related to higher Gensini scores.

	Univariate		Multivariate		
	OR	*P* value	OR	95% CI	*P* value
Lumbar disc herniation	2.23	<0.01^b^	2.38	1.31–4.32	<0.01^c^
Male	1.71	0.06^b^	1.58	0.85–2.93	0.15
Age	1.03	0.13			
BMI^a^	1.00	0.98			
Hypertension	1.28	0.39			
Diabetes mellitus	1.51	0.20			
Drinking history	0.88	0.72			
Smoking history	1	1			
INR^a^	0.62	0.45			
Fibrinogen	1.25	0.15			
D-dimer	1.0	0.3			
ALT^a^	1.01	0.16			
AST^a^	1.02	0.02^b^	1.02	0.99–1.04	0.06
Triglyceride	1.20	0.13			
Total cholesterol	1.05	0.67			
HDL-C^a^	0.41	0.04^b^	0.74	0.28–1.96	0.54
LDL-C^a^	1.14	0.39			
Lipoprotein A	1.00	0.27			
eGFR^a^	0.99	0.13			
Blood glucose	1.24	0.02^b^	1.19	0.98–1.44	0.08
Uric acid	1.00	0.09^b^	1.01	0.99–1.01	0.23
Potassium ion	0.95	0.87			
Sodium ion	0.84	0.01^b^	0.91	0.80–1.02	0.1
LVIDd^a^	1.01	0.98			
LVIDs^a^	1.05	0.85			
LVEF^a^	0.98	0.56			

^a^ BMI, weight and body mass index; INR, international normalized ratio; ALT, alanine aminotransferase; AST, aspartate aminotransferase; HDL-C, high-density lipoprotein cholesterol; LDL-C, low-density lipoprotein cholesterol; eGFR, estimated glomerular filtration rate; LVIDd, left ventricular internal diameter at end-diastole; LVIDs, left ventricular internal diameter at end-systole; LVEF, left ventricular ejection fraction.

^b^ *P* < 0.1, which means variable was considered for inclusion in the multivariate model.

^c^ *P* < 0.05, which means statistically significant in the multivariate model.

### 3.3 Correlation between the degree of lumbar disc lesions and blood lipid levels and Gensini score

Spearman's correlation test (Table 3) suggested that both the Pfirrmann grading system (correlation coefficient = 0.66, *p* < 0.01) and the grading system of migrated LDH (correlation coefficient = 0.73, *p* < 0.01) were positively correlated with the Gensini score. In addition, the Pfirrmann grading system was positively correlated with the LDL-C levels (correlation coefficient = 0.31, *p* = 0.04), while the grading system of migrated LDH was positively correlated with the TG levels (correlation coefficient = 0.32, *p* = 0.04).

**Table 3:** Correlation of the degree of lumbar lesions with Gensini scores and blood lipid levels.

	Pfirrmann grading system		Grading system of migrated lumbar disc herniation	
	Correlation coefficient	*P* value	Correlation coefficient	*P* value
Gensini scores	0.66	<0.01^b^	0.73	<0.01^b^
Triglyceride	0.01	0.99	0.32	0.04^b^
Total cholesterol	0.22	0.18	0.16	0.32
HDL-C^a^	−0.16	0.31	−0.20	0.21
LDL-C^a^	0.31	0.04^b^	0.15	0.35
Lipoprotein A	0.24	0.13	0.01	0.94

^a^ HDL-C, high-density lipoprotein cholesterol; LDL-C, low-density lipoprotein cholesterol.

^b^ *P* < 0.05, which means statistically significant.

### 3.4 One year clinical outcomes

The baseline data of the 56 patients included in the evolocumab treatment group and the conventional treatment group were comparable (Table 4). As shown in Table 5, by 1-year follow-up, there were 42.86% (*n* = 12) composite MACEs in the conventional treatment group and 14.29% (*n* = 4) in the evolocumab treatment group (*p* = 0.04). The incidence of readmission due to angina, underwent nonfatal myocardial infarction, underwent nonfatal stroke, mortality were 21.42% (*n* = 6), 10.71% (*n* = 3), 7.14% (*n* = 2) and 3.57% (*n* = 1) in the conventional treatment group and 7.14% (*n* = 2), 3.57% (*n* = 1), 3.57% (*n* = 1) and 0% (*n* = 0) respectively in the evolocumab treatment group (all *p* > 0.05).

**Table 4:** Baseline indicators and Gensini scores.

	Evolocumab treatment group	Conventional treatment group	*P* value
	(*n* = 28)	(*n* = 28)	
Gensini scores	37.50 (25.63, 51.50)	30.00 (20.00, 47.00)	0.58
ALT^b^ (U/L)	22.25 (14.63, 29.00)	21.04 (14.11, 28.63)	0.91
AST^b^ (U/L)	21.71 (16.00, 28.53)	18.65 (15.40, 31.36)	0.78
Triglyceride (mmol/L)	1.53 (1.10, 2.62)	1.29 (1.00, 1.71)	0.06
Total cholesterol (mmol/L)	4.54 ± 1.20	4.35 ± 1.29	0.53
HDL-C^b^ (mmol/L)	1.03 ± 0.27	1.16 ± 0.37	0.18
LDL-C^b^ (mmol/L)	2.42 ± 0.84	2.51 ± 0.93	0.63
Lipoprotein A (mg/L)	186.00 (97.50, 311.75)	272.00 (121.33, 800.75)	0.18
Aspirin	25 (89.29%)	24 (85.71%)	0.99
P2Y12 inhibitor	22 (71.43%)	18 (64.29%)	0.78
Statins	28 (100%)	28 (100%)	-
Ezetimibe	28 (100%)	25 (89.29%)	0.24

^a^ Data are presented as the mean ± SD, media (*Q*_1_, *Q*_3_) or *n* (%).

^b^ ALT, alanine aminotransferase; AST, aspartate aminotransferase; HDL-C, high-density lipoprotein cholesterol; LDL-C, low-density lipoprotein cholesterol.

**Table 5:** MACEs and percentage changes in laboratory indicators.

	Evolocumab treatment group	Conventional treatment group	*P* value
	(*n* = 28)	(*n* = 28)	
Percentage Change from baseline, %			
ALT^b^	7.78 (−33.15, 103.52)	1.65 (−30.55, 46.28)	0.55
AST^b^	2.24 (−24.30, 37.22)	−5.51 (−31.13, 31.35)	0.48
Triglyceride	−20.13 (−42.21, −3.35)	−16.67 (−36.00, 9.93)	0.30
Total cholesterol	−26.48 ± 20.72	−14.08 ± 21.30	0.06
HDL-C^b^	−1.90 (−12.70, 17.00)	3.27 (−14.66, 25.01)	0.93
LDL-C^b^	−36.08 ± 23.31	−22.92 ± 25.78	0.04^c^
Lipoprotein A	−31.20 ± 24.36	12.10 ± 39.09	<0.01^c^
MACEs	4 (14.29%)	12 (42.86%)	0.04^c^

^a^ Data are presented as the mean ± SD, media (*Q*_1_, *Q*_3_) or *n* (%).

^b^ ALT, alanine aminotransferase; AST, aspartate aminotransferase; HDL-C, high-density lipoprotein cholesterol; LDL-C, low-density lipoprotein cholesterol.

^c^ *P* < 0.05, which means statistically significant.

### 3.5 Subgroup analysis

**Figure 5:**
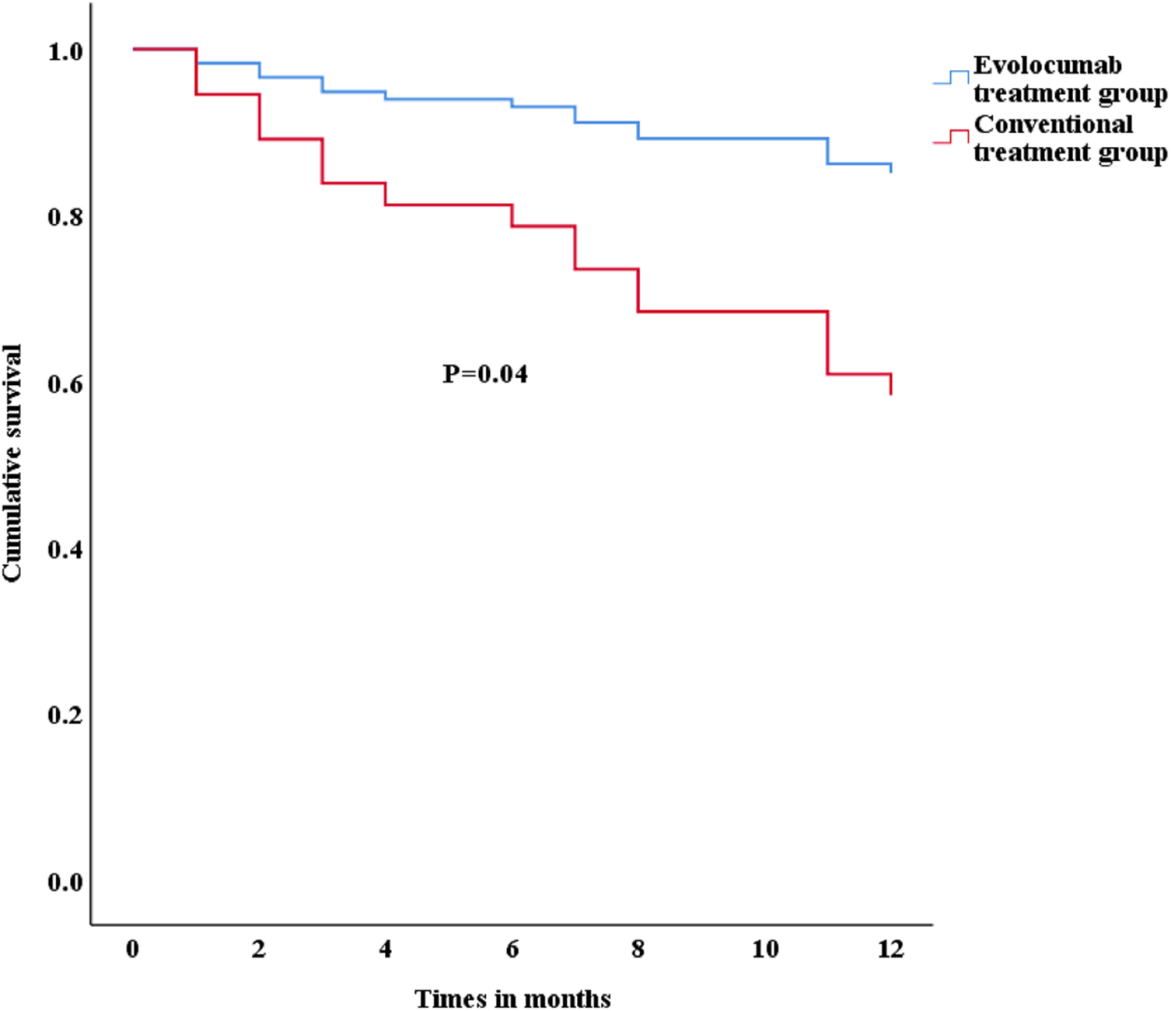
Adjusted survival curve for Cox Proportional Hazards model.

As shown in Table 5, the evolocumab treatment group presented significant reductions in LDL-C (mean: –36.08% VS. −22.92%, *p* = 0.04) and lipoprotein A (mean: −31.2% VS. 12.1%, *p* < 0.01). In addition, as shown in Table 6, the evolocumab treatment (HR = 0.26, 95%CI: 0.07–0.91, *p* = 0.04) significantly influenced the occurrence of MACEs, according to Cox regression analysis. The adjusted survival curve for Cox Proportional Hazards model (Figure 5) showed that patients treated with evolocumab had a better prognosis than those receiving conventional treatment.

**Table 6:** COX regression analysis.

	HR	95% CI	*P* value
Evolocumab treatment	0.26	0.07–0.91	0.04^b^
Percentage change of laboratory indicators			
Triglyceride	0.99	0.97–1.01	0.33
Total cholesterol	1.13	1.02–1.25	0.02^b^
HDL-C^a^	0.95	0.92–0.99	0.01^b^
LDL-C^a^	0.94	0.88–1.01	0.09
Lipoprotein A	0.99	0.97–1.01	0.22

^a^ HDL-C, high-density lipoprotein cholesterol; LDL-C, low-density lipoprotein cholesterol.

^b^ *P* < 0.05, which means statistically significant.

## 4 Discussion

To our best knowledge, this is the first clinical research demonstrated that LDH remained as independent risk factor for coronary plaque burden. Moreover, evolocumab treatment significantly reduced the occurrence of MACEs in LDH patients with higher coronary plaque burden, suggesting LDH increases coronary plaque burden by affecting blood lipid. Although the pathophysiological mechanism is unclear, our data indicate a potential relationship between LDH and coronary plaque burden in UA patients.

### 4.1 Cardiovascular risk factors related to lumbar disc disease

The relationship between cardiovascular risk factors and LDH or degenerative lumbar disc disease attracted increasing attention in recent years. Colombini et al. (16) demonstrated that smoking was correlated with the prevalence of discopathies, osteochondrosis and both associated with LDH. Moreover, Steelman et al. (17) indicated the association between smoking and the development of degenerative disc disease. In addition, Rivinoja et al. (18) found that smoking during adolescence was correlated with hospitalization due to low back pain or sciatica, suggesting smoking as a predictor of LDH. Similar to the previous studies (5), our study indicated the prevelance of positive CAG results (57 vs. 32, *p* < 0.01) was higher in LDH patients. Moreover, our study found that the coronary plaque burden (median Gensini score: 26.5 vs. 14.0, *p* < 0.01) was also higher in LDH group.

### 4.2 Risk factors related to higher coronary plaque burden

Given the important role of coronary plaque burden in atherosclerotic cardiovascular disease risk stratification and management (19), the risk factors related to coronary plaque burden attracted increasing attention in recent years. Won et al. (20) suggested that serum hemoglobin level changes has an independent effect on coronary plaque volume. Akin et al. (21) indicated the coronary plaque burden was positively correlated with non-HDL-C and TG/HDL-C ratio. Moreover, Nafakhi et al. (22) demonstrated that pericardial fat volume independently associated with coronary atherosclerosis burden.

In our study, patients with LDH showed higher TG levels and Gensini scores and lower HDL-C levels. The result suggests LDH as a risk factor for higher coronary plaque burden. We hypothesis LDH increases coronary plaque burden by affecting blood lipid.

### 4.3 Impact of blood lipid levels on outcome

Previous studies by Yuan et al. (23) and Huang et al. (24) have shown that hyperlipidaemia is a risk factor for intervertebral disc degeneration. A study (25) that included 790 patients showed that total cholesterol levels, LDL-C levels, and TG levels were higher in patients with LDH. This may be related to blood lipids directly interfering with the death by pyroptosis of nucleus pulposus cells and damaging extracellular matrix metabolism (26). The latest ESC/EAS guidelines for the management of dyslipidaemias (14) have emphasized the importance of lipid-lowering treatment in reducing cardiovascular risk. Nelson et al. (27) and Nicholls et al. (28) demonstrated that evolocumab treatment can produce coronary plaque stabilization and regression. In this cohort, our results showed that evolocumab treatment significantly reduced occurrence of MACEs in the patients with higher plaque burden. Moreover, our data indicated that LDH is associated with increased blood lipid, which may contribute to the development of plaque burden.

### 4.4 Research significance

Our findings suggested that LDH was an independent risk factor for higher plaque burden in coronary, and blood lipid might play a key role. In LDH patients, controlling cardiovascular risk factors, especially blood lipid, is necessary to avoid coronary atherosclerosis. When considering surgery treatment in patients with LDH, comprehensive assessment of cardiovascular risk is required to avoid cardiovascular events in perioperative period.

### 4.5 Conclusion

LDH is an independent risk factor for the higher coronary plaque burden. Evolocumab treatment significantly reduces the occurrence of cardiovascular events in LDH patients with higher plaque burden. Our research suggests that LDH increases coronary plaque burden by affecting blood lipid.

### 4.6 Limitations

The current study has several limitations. First, it is a retrospective and observational study that could be skewed by confounding variables. Unmeasured factors could not be examined, although we looked at various aspects that potentially influence prognosis. Second, coronary plaque burden was evaluated by CAG, rather than intravascular ultrasound or optical coherence tomography. Due to the limitations of this trial, multicenter, large-sample clinical research is still needed.

## Data Availability

The raw data supporting the conclusions of this article will be made available by the authors, without undue reservation.
